# Captivity-associated variations in fecal testosterone and progesterone metabolite concentrations in mountain gazelle (*Gazella gazella*)

**DOI:** 10.3389/fvets.2025.1621008

**Published:** 2025-09-04

**Authors:** Mina Cansu Karaer, Tolga Kankılıç, Çağatay Tavşanoğlu, Tilen Vake, Alenka Dovč, Tomaž Snoj

**Affiliations:** ^1^Institute of Preclinical Sciences, Veterinary Faculty, University of Ljubljana, Ljubljana, Slovenia; ^2^Department of Biology, Sabire Yazıcı Faculty of Science and Letters, Aksaray University, Aksaray, Türkiye; ^3^Division of Ecology, Department of Biology, Hacettepe University, Ankara, Türkiye; ^4^Clinic for Birds, Small Mammals and Reptiles, Veterinary Faculty, University of Ljubljana, Ljubljana, Slovenia

**Keywords:** gazelle, non-invasive monitoring, reproductive cycle, captivity, dynamics of steroid hormone excretion

## Abstract

**Background:**

Mountain gazelle (*Gazella gazella*) is a medium-sized antelope native to arid regions that is currently listed as an endangered species. There are only two known populations of mountain gazelles worldwide, one of which is in Hatay (Türkiye). In this study, we investigated the differences in the fecal concentrations of testosterone and progesterone metabolites in free-ranging and captive mountain gazelle.

**Methods:**

Fecal samples were collected from the ground in the Hatay Mountain Gazelle Wildlife Development Area, located in Hatay Province (Türkiye) during each season of the year. In total, 246 samples, 170 from free-ranging population and 76 from captive population, were collected and used to determine testosterone and progesterone metabolite concentrations. The metabolites were extracted from dried fecal samples using methanol, and their concentrations were quantified using ELISA. The detection methods were partially validated. The analytical validation includes the determination of coefficients of variation, sensitivity of the measurements, recovery rate, linearity and cross-reactivity. In biological evaluation, the predicted reproductive status of the animals was compared with the concentrations of the progesterone and testosterone metabolites in feces.

**Results and conclusion:**

Our analysis revealed that fecal samples obtained from free-ranging individuals consistently contained higher levels of testosterone metabolites than those obtained from the captive individuals. No consistent pattern was detected for fecal progesterone metabolites. Our results suggest that a constant supply of water stimulates intestinal transit. Therefore, due to faster intestinal transit the population with continuous water availability throughout the year (captive population) has lower concentrations of fecal hormone metabolites. These findings are relevant not only for the mountain gazelle as a species of endangered status but also provide important information regarding the mechanisms underlying the dynamics of steroid hormone excretion in ruminants.

## 1 Introduction

*Gazella gazella* (Antilopini, Bovidae), commonly referred to as the mountain gazelle, is a medium-sized antelope native to arid regions that is currently listed as an endangered species by the International Union for Conservation of Nature (1). Socially, these gazelles often form small herds, and their reproductive strategies can differ depending on environmental conditions (2). This species is generally considered a seasonal breeder, with the onset of the estrous cycle in the studied population, occurring primarily in December and January, and the fawning season typically occurring between May and June. Following birth, the subsequent months of June to August are characterized by lactation, which continues for approximately 3 months, and, thereafter, between September and November, the females are anestric (3). In seasonal breeders, the end of the anestrus phase coincides with an activation of the hypothalamic–pituitary-gonadal (HPG) axis, triggered by environmental factors such as daylight length, which determine the timing of reproductive cycles, thereby ensuring optimal conditions for offspring survival (4).

The reproductive functions and sexual behavior of mammals are coordinated by the HPG axis (5), with the hormones testosterone, progesterone, and estrogen playing essential roles in the regulation of reproductive functions (6). Among these, progesterone, which plays prominent roles in the estrous cycle and pregnancy, contributes to maintaining a stable environment for fetal development (7). It also promotes mammary gland development and has a pronounced influence on central nervous system and cardiovascular function (6). Notably, although it serves as a key hormone in female reproduction, progesterone also plays an important role in males, in which it contributes to the regulation of spermiogenesis and functions as a precursor of testosterone biosynthesis in Leydig cells (8). Furthermore, as a neurosteroid, progesterone has been established to be involved in the sleep–wake cycle (9). In contrast, testosterone plays vital roles in the development of male characteristics and reproductive functions, including spermatogenesis and spermiogenesis (10), and also has anabolic effects, thereby contributing to muscle and bone growth. Moreover, testosterone stimulates mating drive and influences the regulation of mood (11). However, although generally considered a male hormone, testosterone also plays important roles in females, influencing ovulation and behavior during estrus. Importantly, during steroidogenesis in the ovaries and placenta, testosterone is aromatized and metabolized to 17β-estradiol (8, 12, 13). Collectively, these hormones are integral not only to reproductive health but also to the overall physical and emotional wellbeing of individuals, thereby highlighting their importance with respect to both male and female physiology (14).

Having served their primary purpose in organisms, steroid hormones such as testosterone and progesterone are metabolized in the liver by CYP450 microsomal enzymes and excreted via bile and urine (15, 16). Hormone metabolites excreted through bile enter the intestinal tract and are eventually eliminated with feces, making fecal hormone analysis possible. In this regard, determining the concentrations of fecal hormone metabolites has the advantages of being a simple and non-invasive sampling strategy. Consequently, a knowledge of the excretion dynamics of hormone metabolites is important with respect to interpreting the physiological processes that are reflected in the concentrations of fecal hormone metabolites.

However, gaining an understanding these mechanisms in different species, particularly in those of conservation significance, requires species-specific hormonal studies. To date, several such studies have been conducted on gazelle species to investigate the dynamics of reproductive hormones, including *G. dorcas* (17), *G. dama mhorr* (18, 19), *G. subgutturosa subgutturosa* (20), *G. gazella* (21), and *G. subgutturosa marica* (20, 22). However, the effects of population status on testosterone and progesterone levels in the different sexes among the captive and free-ranging populations of these species have yet to be thoroughly investigated.

On the basis of the finding of studies that have revealed differences in glucocorticoid secretion in the captive and free-ranging populations of several species (3, 23), we wanted to assess differences in the fecal concentrations of testosterone and progesterone metabolites in free-ranging and captive mountain gazelles. Given that the microenvironmental conditions of these two populations differ in terms of water supply and nutrition, we anticipated that these differences would be reflected in the fecal concentrations of testosterone and progesterone metabolites. Our findings in this study elucidate the dynamics of sex hormone metabolite excretion in mountain gazelle, and the fact that there are only two known populations of mountain gazelles worldwide (3, 24), one of which is Hatay (Türkiye), highlights the significance of these findings.

## 2 Materials and methods

### 2.1 Study area

**Figure 1:**
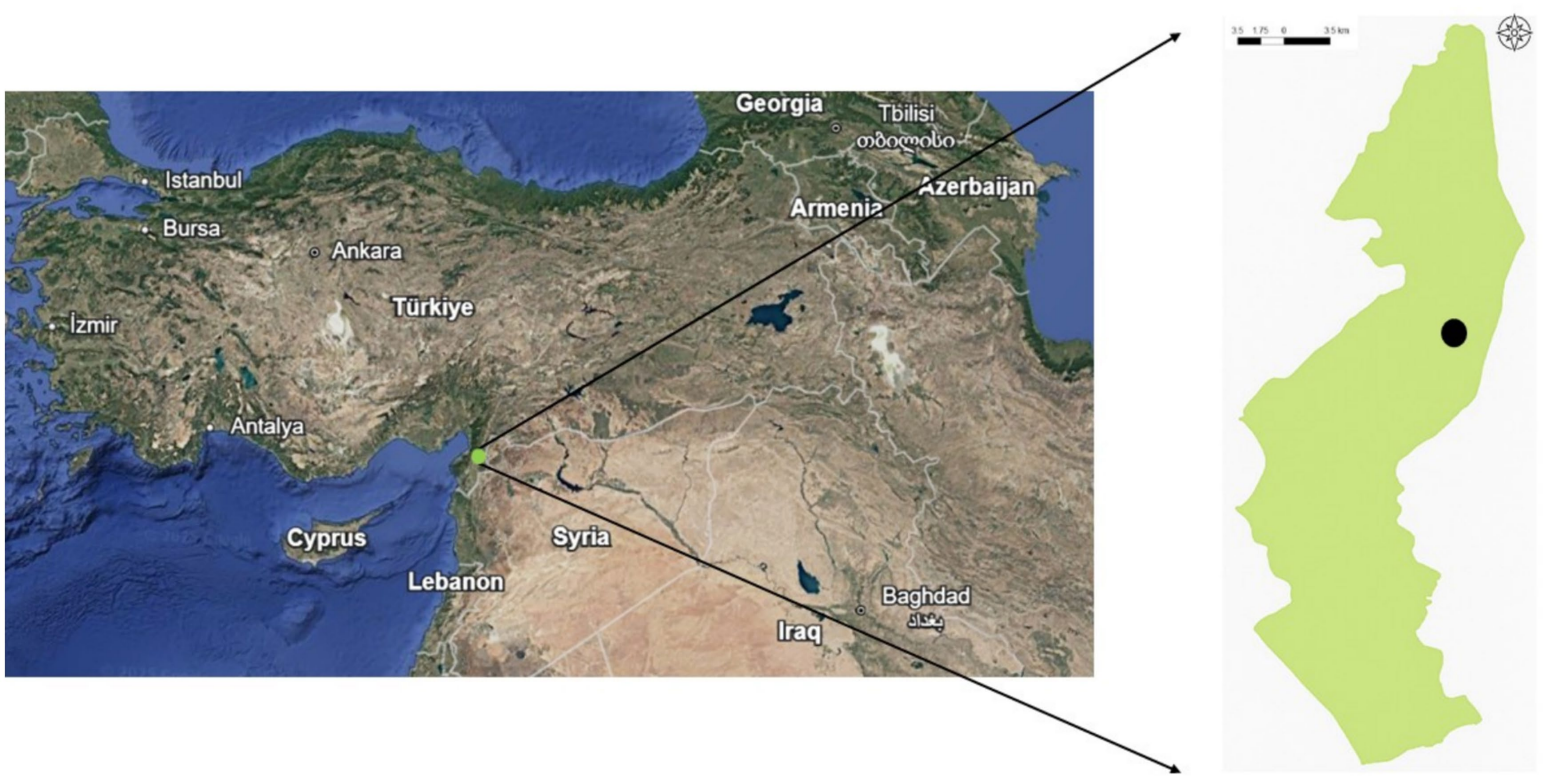
Map of Türkiye showing the Hatay Mountain Gazelle Wildlife Development Area highlighted in green. The black dot within the map indicates the location of the Hatay Mountain Gazelle Production Center.

**Figure 2:**
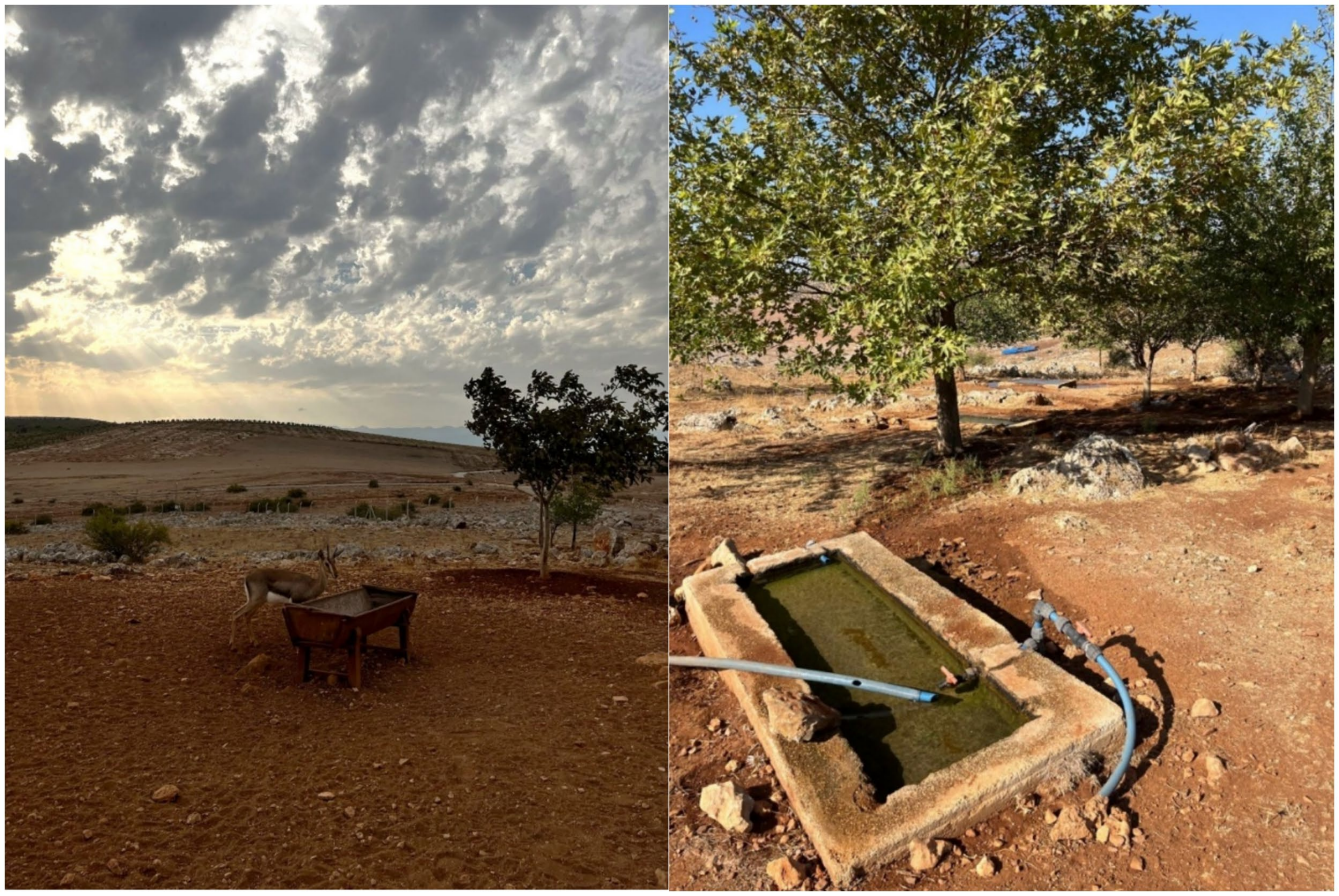
On the left: An individual from the captive population feeding throughout the year from a provided feeder. The fences enclosing the Hatay Mountain Gazelle Production Center are visible behind the animal, and beyond the fence lies the area inhabited by the free-ranging population and other wild species. On the right: A year-round water source provided for the captive population within the boundaries of the Production Center.

This study was conducted in the Hatay Mountain Gazelle Wildlife Development Area, located near the Syrian border in Hatay Province, Türkiye (36°32′N, 36°32′E; elevation 200–450 m, Figure 1). The region in which the study was conducted encompasses an area of 13,228 hectares, consisting of grassland vegetation and shrublands, extensive agricultural land, and rocky hills. The study area is characterized by a warm climate, with mild winters and hot, dry summers with no recorded precipitation. Spring and autumn are transitional periods with moderate temperatures and low rainfall, reflecting semi-arid climatic conditions (3, 25). As of 2025, the population of mountain gazelles in this area is estimated to be 1,504 individuals (data from Nature Conservation and National Parks, Hatay Branch), whereas at the time this study was conducted in 2023 in this region, the estimated population stood at 1,387. This habitat, which supports a diverse range of mammalian fauna, is actively utilized by the gazelle throughout the year and represents the northernmost distribution of the species and is the only known habitat of mountain gazelles in Türkiye (26). Notably, the protected area contains both free-ranging and captive populations of mountain gazelles that accordingly experience similar macroenvironmental conditions, including photoperiods and climatic conditions. Moreover, the areas inhabited by both free-ranging and captive populations are characterized by a similar vegetation (Figure 2). However, whereas animals in the captive population have constant access to barley, hay, and water supplied by the staff of the Direction of Nature Conservation and Natural Parks of Hatay (Figure 2), in the case of the free-ranging population, access to water tends to be limited. Captive population is housed within a 12-hectare fenced area at the Hatay Mountain Gazelle Production Center, located inside the study region (3, Figure 1). At the time of sampling, the population consisted of 39 females and 11 males. The Production Center was originally established to support the small local population and to facilitate reintroduction efforts, though this goal has not yet been realized. It also provides care for injured individuals and orphaned newborns. Due to habituation to humans, these animals are not released back into the wild. The population breeds within the enclosure, and offspring remain with their parents. Veterinary interventions are limited to essential cases such as injury or illness. As such, the management conditions differ considerably between the free-ranging and captive populations. For the purpose of this study, we use the term “population status” to indicate whether a population is captive or free-ranging.

### 2.2 Sample collection and measurement of hormone metabolites in fecal samples

Fecal samples were collected from the ground in the natural habitat of mountain gazelles from both free-ranging and captive unknown individuals. Prior to fecal sample collection, mountain gazelles were observed at a minimum distance of 500 m for free-ranging individuals and 50 m captive individuals. Gazelles tend to form groups during certain periods, while at other times some individuals may remain solitary (3). During our observations, when an individual or group was seen, the area was promptly approached either on foot or by vehicle, and the freshest fecal samples were collected as quickly as possible. Approximately 7 g of feces were gathered from each sample, placed into polypropylene tubes, and kept at 4 °C in a cooler during transport to the laboratory. Although animals were monitored closely before sample collection, it was not possible to assign fecal samples to specific individuals. As a result, the sex and age of the animal that produced each sample remain unknown. Furthermore, mountain gazelles, particularly those in free-ranging populations, are highly vigilant and are likely to withdraw when encountering unfamiliar stimuli or anthropogenic activity. This behavior, observed in both wild and captive populations, limited our ability to achieve individual-level identification at the time of non-invasive sampling. Nevertheless, based on direct observation of defecation events and spatiotemporal separation during sampling, we assumed that each fecal sample originated from a different individual. Sample collection months were selected to represent biologically relevant reproductive periods (i.e., mating in December, gestation in April, lactation in July, and non-reproductive in September), which also correspond to calendar seasons (winter, spring, summer, and autumn, respectively). This alignment allowed for evaluation of both reproductive status and potential seasonal variation in hormone levels.

The fecal samples were stored in separate plastic bags, labeled with the time, date, and location of collection and maintained at −20 °C until used for analysis. The analysis of hormone metabolite concentrations began with drying the samples at 50 °C for 24 h. Thereafter, each dried sample was ground using a mortar and pestle, and then 0.5 g of dried fecal powder was transferred to tube to which 5 mL of 80% methanol was added. Following vortexing for 15–20 s, the samples were extracted for 30 min with shaking, after which, they were centrifuged for 15 min at 3,500×*g*. After centrifugation, 100 μL of each extract was pipetted into two Eppendorf tubes (one for progesterone metabolites determination and other for testosterone metabolites determination) and 900 μL of phosphate-buffered saline (PBS) was added to each tube (first dilution step). The diluted extracts were vortexed and stored at −20 °C until progesterone and testosterone metabolite concentrations were determined by ELISA (within one to two days). After ELISA, diluted extracts were kept at −20 °C.

Two hours before ELISA analysis, the diluted extracts were taken from the freezer and thawed at room temperature. ELISA was performed using a Progesterone ELISA kit (DE 1561; Demeditec, Kiel, Germany) and a Testosterone ELISA kit (DE 1559; Demeditec, Kiel, Germany), with concentrations in the fecal extracts being measured in duplicate, following the manufacturer’s instructions, and absorbance measured at 450 nm using a Multiskan FC microtiter plate reader (Thermo Fisher Scientific, Waltham, United States). The results obtained for progesterone and testosterone metabolite concentrations in terms of nanograms per milliliter of extract were converted to nanograms per gram of dry feces. The diluted extracts (first dilution step) containing concentrations of progesterone metabolites that exceeded the detection limit of the ELISA (4,000 ng/g) were further diluted (100 μL in 900 μL PBS—second dilution step) and determined again.

As partial validation of the measurements, we determined intra- and inter-assay coefficients of variation (CV), sensitivity, recovery, linearity and stated cross-reactivity provided by manufacturer. To determine CVs for the progesterone test, two fecal extracts were measured 10 times in a single assay and four times in each additional assay, whereas for testosterone, the two fecal extracts were measured four times in one assay and four times in each additional assay. For progesterone metabolites, we accordingly obtained intra-assay CV values of 8.27 and 12.11% for samples containing 1980 and 2,155 ng/g metabolites, respectively, whereas the corresponding inter-assay CV values for the same samples were 8.18 and 16.05%. Similarly, for testosterone metabolites, intra-assay CV values of 7.45 and 5.78% were obtained for samples containing 195 and 710 ng/g testosterone metabolites, respectively, with corresponding inter-assay CVs of 4.66 and 10.06%.

The sensitivity of the assay for detecting fecal progesterone and testosterone metabolites is reflected in the detection ranges of the respective kits. For progesterone metabolites, the detection range was 40–4,000 ng/g when using fecal extracts with the initial (first) dilution step. Samples with concentrations exceeding 4,000 ng/g, were subjected to an additional (second) dilution, extending the measurable range to 400–40,000 ng/g. Overall, the combined detection range for fecal progesterone metabolites was 40–40,000 ng/g. For testosterone fecal metabolites, the detection range of the assay measurements was 20–1,600 ng/g.

Recovery rates were assessed using dried fecal samples with previously determined concentrations of progesterone and testosterone metabolites. Aliquots of 0.5 g of dry fecal samples were spiked with 1,500 ng of progesterone standard and either 100 ng and 500 ng of testosterone standard (both from Sigma-Aldrich, St. Louis, USA), previously diluted in methanol. After spiking, the samples were subjected to the same methanol extraction protocol as described above. Recovery was calculated comparing the measured hormone concentrations to the expected values, yielding recovery rates of 113% for progesterone and 96 and 115% for testosterone, respectively.

Linearity was assessed using two fecal samples: one with previously determined high concentration of progesterone and the other with a high concentration of testosterone metabolites. Fecal extracts from both samples were serially diluted with PBS in a stepwise 1:1 ratio. The results are presented in Table 1.

**Table 1:** Results of the linearity test for progesterone and testosterone measurements.

Dilution	Fecal progesterone metabolites		Fecal testosterone metabolites	
	Expected concentration (ng/g)	Measured concentration (ng/g)	Expected concentration (ng/g)	Measured concentration (ng/g)
Undiluted	3,525		262	
1:2	1,762	2,103	131	156
1:4	881	774	65.5	72
1:8	440	338	32.75	41,5
1:16	220	162	16.37	21
1:32	110	136	8.19	15

The coefficients of determination (R^2^) were 0.986 for progesterone metabolite and 0.992 for testosterone metabolite measurements.

Details of the cross-reactivity of the ELISAs with other steroids were provided by the manufacturer. The cross-reactivities of the progesterone ELISA test are as follows: pregnenolone 0.35%; 17α OH progesterone 0.3%; corticosterone 0.2%; 11-deoxycorticosterone 1.1%; and individual natural estrogens, testosterone, and other glucocorticoids ≤0.1%. The cross-reactivities of the testosterone ELISA are as follows: dihydrotestosterone 12.9%; 11β-hydroxytestosterone and 19-nortestosterone 3.3%; androstenedion 0.9%; 5α-dihidrotestosterone 0.8%; and epitestosterone, progesterone, cortisol and individual natural estrogens <0.1.

Biological evaluation was only partially performed, as the exact age, reproductive status and health condition of individual animals were unknown. However, it is likely that most females were pregnant in April and lactating in July, based on the species’ reproductive biology. The observed high fecal progesterone metabolite concentrations in April and low concentrations in July are consistent with this expected physiological pattern and support biological relevance of the assay. Furthermore, we found a significant positive correlation between the concentrations of testosterone and progesterone fecal metabolites as detailed in the Results section. A positive correlation between testosterone and progesterone has been described previously (27, 28), so our results showing this phenomenon can be considered as evidence of a correct method of analysis. Collectively, these results provide partial biological validation of the applied method.

### 2.3 Sex determination

The sex of the animals was determined by detecting the *SRY* gene in the DNA extracted from fecal samples, as described previously (3). Of the 246 fecal samples analyzed, 125 were assessed to be derived from females and 121 were from males. These results are in line with the expected proportions and enabled us to determine the composition of the studied animal groups, as shown in Table 2.

**Table 2:** Number of fecal samples collected from captive (C) and free-ranging (FR) gazelles.

Samples	Dec-22		Apr-23		Jul-23		Sep-23	
	C	FR	C	FR	C	FR	C	FR
Female	2	19	6	14	13	28	14	29
Male	7	11	14	14	12	24	8	31
Total	9	30	20	28	25	52	22	60

### 2.4 Statistical analyses

Data analysis was performed with R^1^ using the *stats* package for model fitting, *emmeans* for *post hoc* comparisons, and *ggplot2* for data visualization. Fecal testosterone and progesterone metabolite concentrations were analyzed using generalized linear models (GLMs) with a gamma distribution and a log-link function suitable for modeling continuous positive data with a skewed distribution. Each hormone was modeled using a three-way interaction between sex, population status, and season as follows:


Progesterone~Sex×Population_status×Season

and


Testosterone~Sex×Population_status×Season.

Model selection was based on the lowest Akaike’s information criterion (AIC) and the lowest residual deviance compared with those of simpler models based on two-way interactions or only main effects. Model adequacy was assessed by examining the quantile residuals, and influential observations were identified using Cook’s distance and leverage values. Observations with unusually high values, particularly those with leverage exceeding twice the average were flagged for further inspection. Data points deemed physiologically implausible or overly influential were excluded prior to refitting the model. Model fit was assessed using McFadden’s and Nagelkerke’s pseudo-R^2^, along with the AIC values.

Pairwise comparisons were performed to assess the differences between captive and free-ranging individuals within each sex and season using the estimated marginal means. Sidak’s correction, which maintains statistical power while adjusting for the family-wise error rate, was applied to control for multiple comparisons. Owing to the log-link function, all comparisons were performed using a logarithmic scale. The estimates were back-transformed by exponentiation, and the results are reported as multiplicative effects (ratios) rather than absolute differences in logarithms. The correlation between fecal progesterone metabolite and testosterone metabolites concentrations was assessed using Pearson’s correlation coefficients. To account for seasonal and sex-specific variations, analyses were conducted separately for each combination of season and sex. For tests, the statistical significance was set at *p* < 0.05.

## 3 Results

Model selection for fecal testosterone and progesterone metabolite concentrations was conducted by comparing different GLMs using the AIC and deviance as measures of fit (Table 3). In both cases, a model including the three-way interaction provided the best fit, indicating that the interplay among sex, population status, and season is important for explaining the detected variability in hormone concentrations.

**Table 3:** Parameters of model selection used to describe fecal testosterone and progesterone metabolite concentrations in *Gazella gazella.*

Model equations	AIC	Deviance
Progesterone		
*~ Sex + Population status + Season*	3,711	194.3
*~ Sex + Population status × Season*	3,704	185
*~ Sex × Population status + Season*	3,710	192.6
*~ Sex × Season + Population status*	3,706	186.7
***~ Sex × Population status × Season***	**3,683**	**162.8**
Testosterone		
*~ Sex + Population status + Season*	2,935	44.3
*~ Sex + Population status × Season*	2,931	42.6
*~ Sex × Population status + Season*	2,936	44.1
*~ Sex × Season + Population status*	2,933	43.0
***~ Sex × Population status × Season***	**2,926**	**39.5**

The final models selected are highlighted.

To ensure more conservative analyses, values considered as influential outliers were excluded prior to fitting the final models. All these outliers were at the high end of the concentration range and were obtained for samples derived from females in the free-ranging population (Table 4).

**Table 4:** A list of identified outliers in the data that were excluded prior to model fitting.

Sex	Population status	Season	Hormone	Concentration (ng/g)
Female	Free-ranging	December	Progesterone	38,000
Female	Free-ranging	April	Progesterone	33,200
Female	Free-ranging	April	Progesterone	40,000
Female	Free-ranging	April	Progesterone	40,000
Female	Free-ranging	April	Progesterone	40,000
Female	Free-ranging	September	Progesterone	19,550
Female	Free-ranging	December	Progesterone	32,200
Female	Free-ranging	December	Testosterone	1,260
Female	Free-ranging	September	Testosterone	1,030
Female	Free-ranging	April	Testosterone	1,020

### 3.1 Testosterone

The descriptive statistics pertaining to data obtained for the fecal testosterone metabolites are shown in Table 5. In the analysis, we identified three measurements as outliers, which were accordingly removed prior to model fitting (Table 4).

**Table 5:** Characteristics of fecal samples included in the final analysis, with distribution presented according to population status, sex, season, with the mean and range of testosterone metabolite concentrations.

Population status	Sex	Season	No. of animals	Mean (ng/g)	Range (ng/g)
Captive	Female	December	2	145.0	135–155
		April	6	237.0	165–376
		July	13	147.2	90–223
		September	14	218.2	128–408
	Male	December	7	134.9	110–181
		April	14	277.9	177–441
		July	12	161.6	92–237
		September	8	173.1	96–300
Free-ranging	Female	December	18	289.2	137–772
		April	13	549.8	235–880
		July	28	241.6	100–571
		September	28	213.8	103–434
	Male	December	11	275.0	109–409
		April	14	340.9	126–675
		July	24	271.0	140–574
		September	31	279.0	114–779

**Figure 3:**
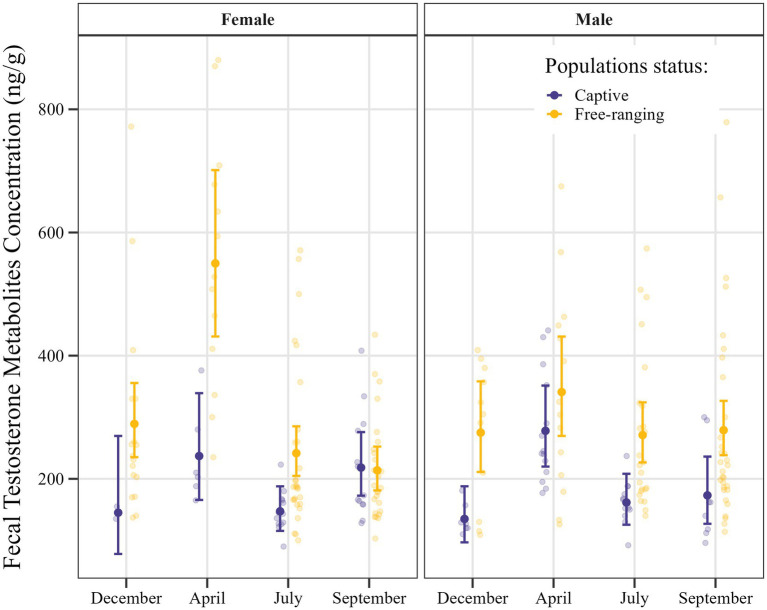
Estimated fecal testosterone metabolite concentrations in *Gazella gazella*, shown in terms of population status and sex, with 95% confidence intervals.

The GLM applied for analysis of fecal testosterone metabolite concentrations utilizing a gamma distribution was found to have moderate explanatory power, as indicated by Nagelkerke’s pseudo-R^2^ value of 0.39, whereas the value obtained for McFadden’s pseudo-R^2^ was lower at 0.038. The fit of the model, as indicated by the AIC, was 2,926, with null and residual deviance values of 62.3 and 39.5, respectively. A summary of the model estimates and their confidence intervals is provided in Supplementary Table A, with the model-predicted values being shown in Figure 3.

*Post hoc* comparisons of fecal testosterone metabolite concentrations in samples obtained from captive and free-ranging individuals of *G. gazella* among seasons and in both sexes are shown in Table 6. Overall, we established that the feces derived from free-ranging individuals contained consistently higher and more variable concentrations of testosterone metabolites than the samples obtained from captive individuals.

**Table 6:** Pairwise comparisons of fecal testosterone metabolites in free-ranging (FR) and captive (C) *Gazella gazella* in different seasons.

Population status comparison	Season	Sex	Ratio	SE	Lower CI	Upper CI	t-ratio	*p*-value
FR/C	December	F	1.99	0.66	1.04	3.84	2.08	0.039
	April	F	2.32	0.51	1.51	3.58	3.83	< 0.001
	July	F	1.64	0.25	1.22	2.20	3.32	0.001
	September	F	0.98	0.14	0.74	1.31	−0.14	0.888
FR/C	December	M	2.03	0.44	1.33	3.11	3.31	0.001
	April	M	1.22	0.21	0.88	1.71	1.21	0.226
	July	M	1.67	0.26	1.23	2.29	3.29	0.001
	September	M	1.61	0.28	1.14	2.28	2.70	0.007

The results are shown separately for female (F) and male (M) animals.

SE, standard error.

CI, 95% confidence interval.

With respect to females, we detected significant differences among the samples collected in December, April, and July, when free-ranging individuals had approximately 2.0- (*p* = 0.039), 2.3- (*p* < 0.001), and 1.6-fold (*p* = 0.001) higher testosterone metabolite levels, respectively. Moreover, seasonal variation was established to be more pronounced in free-ranging females, peaking in April, whereas in captive females, concentrations tended to relatively stable across the seasons.

For males, we detected similarities between the free-ranging and captive populations with respect to seasonal patterns in testosterone metabolite concentration, free-ranging males were found to have consistently higher concentrations throughout the year, which were 2.0-, 1.67-, and 1.61-fold higher in December (*p* = 0.001), July (*p* = 0.001), and September (*p* = 0.007), respectively.

### 3.2 Progesterone

Our analysis of fecal progesterone metabolite concentrations revealed seven measurements deemed to be outliers, which were accordingly removed prior to model fitting. Descriptive statistics for the remaining fecal progesterone metabolites data are presented in Table 7.

**Table 7:** Characteristics of fecal samples included in the final analysis, with distribution presented according to population status, sex, season, with the mean and range of progesterone metabolite concentrations.

Population status	Sex	Season	No. of animals	Mean (ng/g)	Range (ng/g)
Captive	Female	December	2	2,235.5	471–4,000
		April	6	1,665.8	576–4,000
		July	13	227.2	52–639
		September	14	1,020.3	257–3,525
	Male	December	7	404.1	187–638
		April	14	1,915.4	703–4,000
		July	12	357.4	67–803
		September	8	216.5	113–371
Free-ranging	Female	December	17	2,268.8	510–7,450
		April	10	1,936.2	247–4,000
		July	28	775.6	109–3,393
		September	28	534.1	170–4,280
	Male	December	11	987.8	264–2,621
		April	14	1,716.2	330–3,648
		July	24	662.0	163–6,275
		September	31	761.7	169–3,725

**Figure 4:**
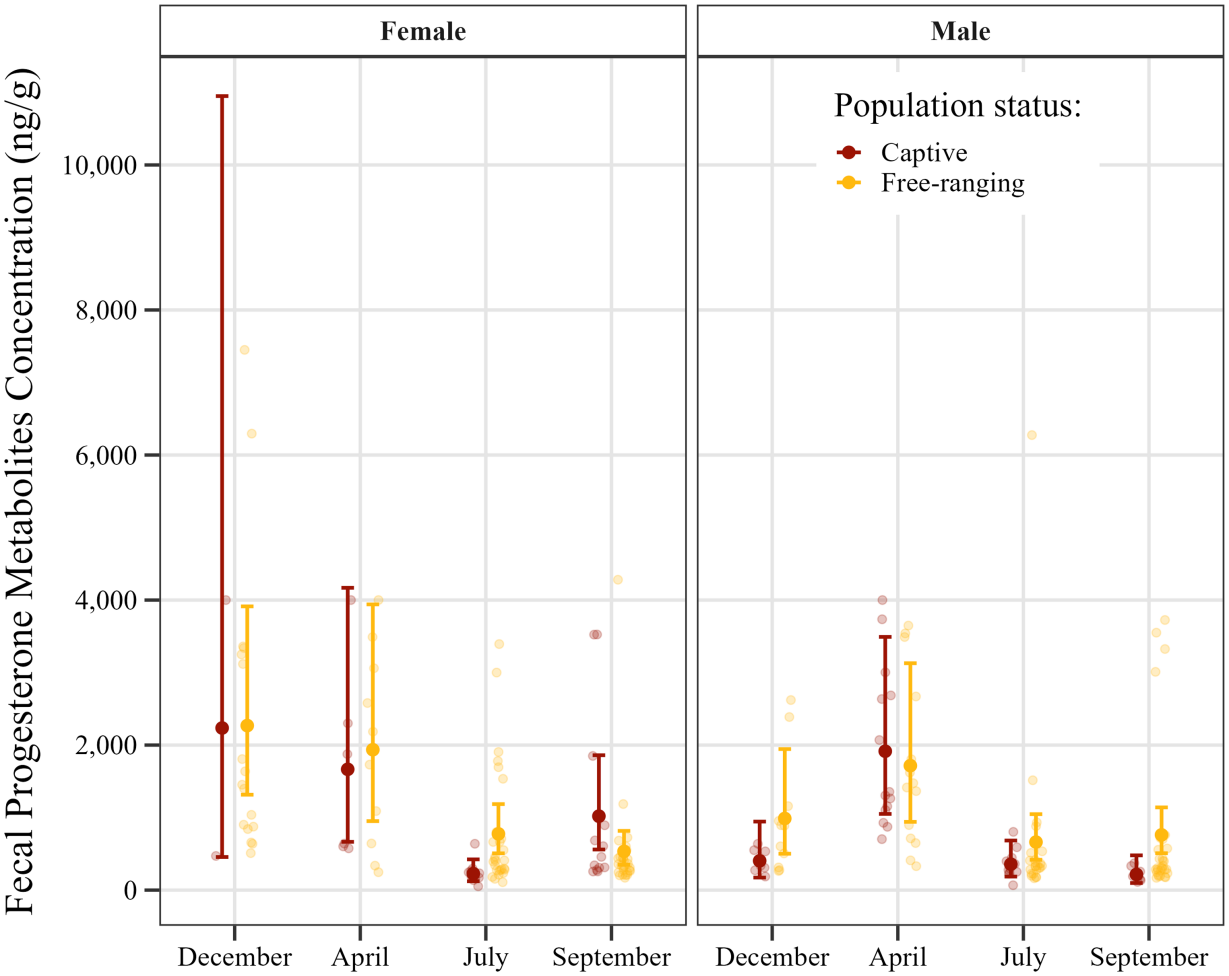
Seasonal distribution of estimated fecal progesterone metabolite concentrations in *Gazella gazella* in terms of population status and sex, with 95% confidence intervals.

The GLM developed for analysis of fecal progesterone metabolite concentrations using a gamma distribution was found to have moderate explanatory power, as indicated by a Nagelkerke’s pseudo-R^2^ value of 0.50, whereas McFadden’s pseudo-R^2^ was lower at 0.034. Model fit as assessed using the AIC was 3,683, and the null and residual deviances of the model were 259.9 and 162.8, respectively. A summary of the model estimates and confidence intervals can be found in Supplementary Table A, with the model-predicted values being shown in Figure 4.

*Post hoc* comparisons of progesterone metabolite concentrations in samples obtained from captive and free-ranging populations across seasons and for both sexes are presented in Table 8. Overall, we detected comparable concentrations of progesterone metabolites in samples derived from males and females, although we noted a distinct seasonal pattern. In females, concentrations were elevated from December through April, followed by a decline in July and September. Contrastingly, for males we detected a single peak in April, whereas levels were relatively stable and lower in the remaining months.

**Table 8:** Seasonal pairwise comparisons of fecal progesterone metabolites between free-ranging (FR) and captive (C) *Gazella gazella*.

Population status comparison	Season	Sex	Ratio	SE	Lower CI	Upper CI	t-ratio	*p*-value
FR/C	December	F	1.01	0.86	0.18	5.44	0.02	0.986
	April	F	1.16	0.68	0.36	3.709	0.26	0.798
	July	F	3.41	1.3	1.6	7.26	3.21	0.002
	September	F	0.52	0.19	0.25	1.09	−1.73	0.084
FR/C	December	M	2.44	1.34	0.82	7.24	1.62	0.106
	April	M	0.89	0.38	0.38	2.09	−0.25	0.799
	July	M	1.85	0.74	0.83	4.09	1.53	0.127
	September	M	3.51	1.59	1.44	8.58	2.78	0.006

The results are shown separately for female (F) and male (M) animals.

SE, standard error.

CI, 95% confidence interval.

Furthermore, a comparison with respect to population status revealed differences in certain seasons. Although progesterone metabolite levels were generally low in July, the concentrations in samples obtained for free-ranging females were 3.4-fold higher than those in the samples obtained for captive females (*p* = 0.002). Similarly, in September, concentrations in samples derived from free-ranging males were 3.5-fold higher than those in samples from captive males (*p* = 0.006). Moreover, throughout the year, free-ranging individuals of both sexes showed a wider dynamic range of progesterone metabolite levels than the captive individuals.

It is important to note that only two samples were obtained from captive females in December, thereby contributing to highly uncertain model estimates. The wide confidence intervals observed for this group reflect this limitation and should accordingly be interpreted with caution.

### 3.3 Correlation analysis

**Figure 5:**
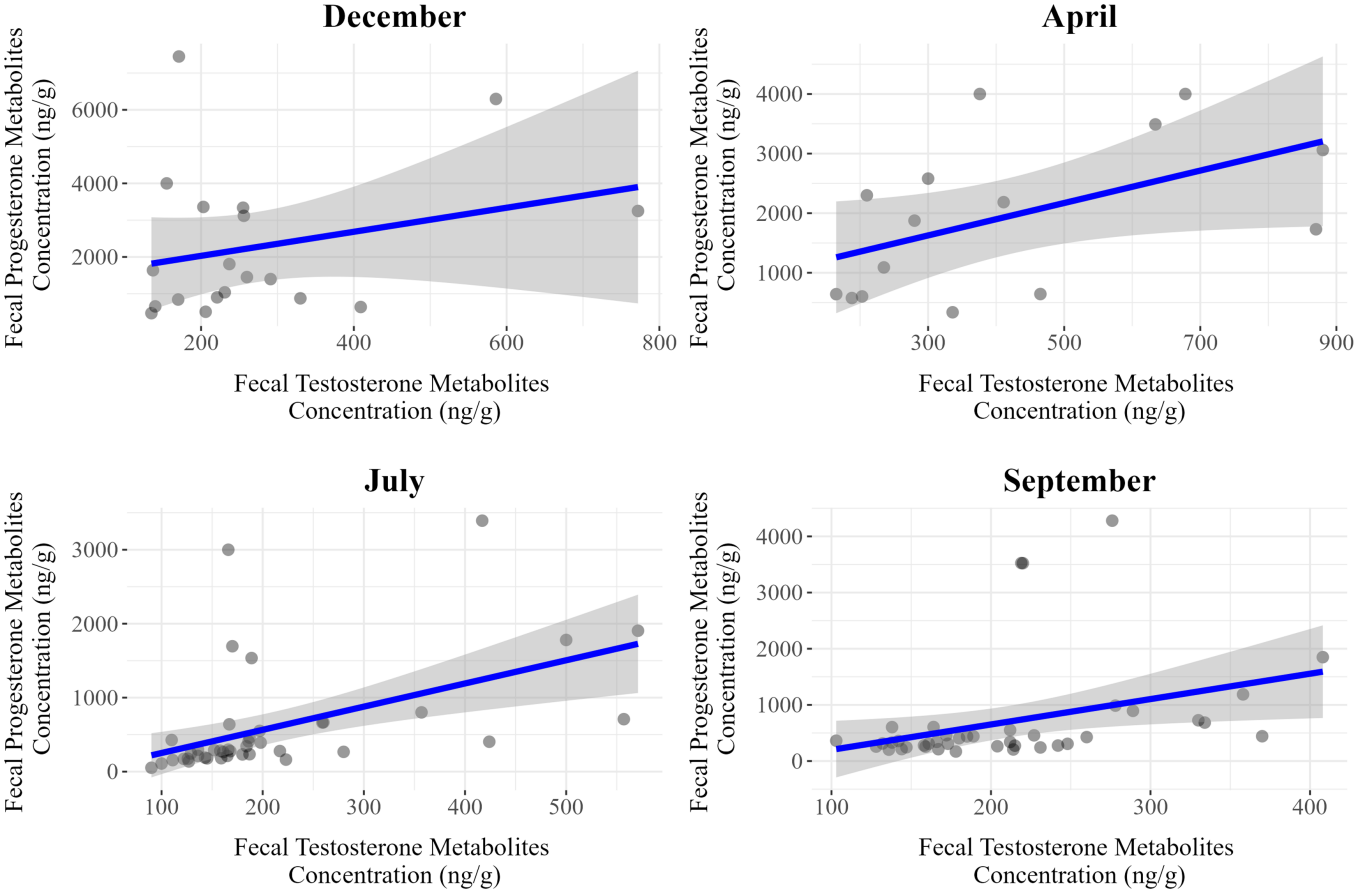
Seasonal correlations between fecal progesterone and testosterone metabolite concentrations in female *Gazella gazella.*

**Figure 6:**
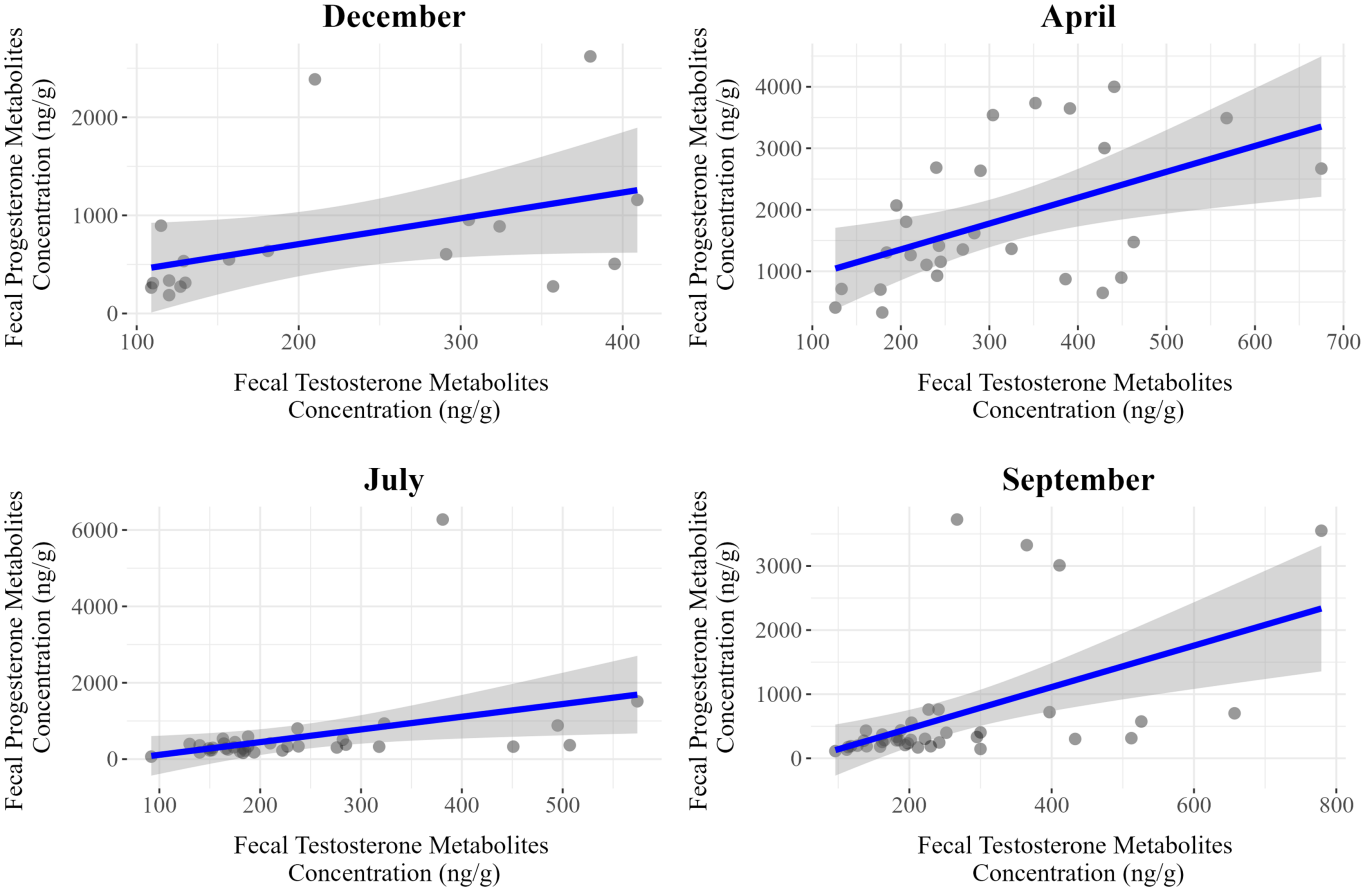
Seasonal correlation between fecal progesterone and testosterone metabolite concentrations in male *Gazella gazella.*

The dataset used for analyses of the correlations between fecal progesterone and testosterone metabolite concentrations was the same as that used for modeling, with the previously identified outliers excluded. The results obtained for separate evaluations performed for each season and sex are summarized in Table 9 and illustrated in Figure 5 for females and Figure 6 for males.

**Table 9:** Pearson correlation coefficients for the seasonal and sex-specific associations of fecal progesterone and testosterone metabolite concentrations in *Gazella gazella.*

Sex	Season	Correlation (r)	Lower CI	Upper CI	*p*-value
Female	December	0.267	−0.213	0.643	0.269
Female	April	0.511	−0.001	0.811	0.051
Female	July	0.500	0.228	0.700	<0.001
Female	September	0.350	0.048	0.594	0.025
Male	December	0.432	−0.044	0.748	0.074
Male	April	0.500	0.156	0.736	0.007
Male	July	0.378	0.057	0.629	0.023
Male	September	0.512	0.235	0.713	<0.001

CI, 95% confidence interval.

Among females, we detected moderate positive correlations in July (*r* = 0.50, *p* < 0.001) and September (*r* = 0.35, *p* = 0.025), whereas weaker associations were observed in December (*r* = 0.27, *p* = 0.269) and April (*r* = 0.51, *p* = 0.051). Contrastingly, for males, there were positive correlations in all seasons, with stronger associations being detected in April (*r* = 0.50, *p* = 0.007), July (*r* = 0.38, *p* = 0.023), and September (*r* = 0.51, *p* < 0.001). Thus, the strongest associations in males and females were apparent in September and July, respectively.

## 4 Discussion

In this study, we sought to evaluate the influence of microenvironmental conditions on fecal hormone excretion based on comparisons of the levels of fecal testosterone and progesterone metabolites in free-ranging and captive mountain gazelles. Our findings revealed a clear pattern, with significantly lower concentrations of testosterone metabolites being detected in the feces of captive individuals than in those of the free-ranging animals (Figures 3, 4). Previous studies in this respect have provided similar evidence indicating that captivity can significantly alter testosterone levels, often resulting in reduced concentrations and disrupted seasonal hormonal cycles compared to free-ranging animals (29–31). Given that this significant difference in testosterone metabolite concentrations was detected in both sexes in the respective populations, we speculate that certain microenvironmental factors may have an influence. In this regard, although we were unable to comprehensively evaluate the effects of different environmental factors during fieldwork, differences in water availability and nutrition were noted. It should, nevertheless, also be highlighted that observed hormonal differences between the two populations could to a large extent be attributable to a high variability in the values obtained, with notably higher levels in a few individuals skewing the mean values in the free-ranging population (Figures 3, 4). To counter this effect to some extent, certain data points considered outliers were excluded from statistical analyses. Notably, all of these outliers were high values obtained for samples derived from female individuals in the free-ranging population (Table 4). These findings accordingly indicate that although the outlier data obtained for these individuals were excluded from the statistical analyses, they do, nonetheless, highlight the natural variability within the free-ranging population in this regard. However, even given this variability, we should not necessarily dismiss the contribution of environmental factors in determining the differences between the two populations.

In contrast to the captive individuals, which had continuous ad libitum access to water, we noted a lack of available water in the habitat used by the free-ranging population. In this regard, it has been reported that the intestinal passage is influenced by the frequency of water consumption (19, 32, 33), and thus a limited water intake can contribute to retarding the intestinal passage, thereby leading to a longer retention of the digesta within the digestive tract. Moreover, the prolonged presence of digesta in the intestines of ruminants is associated with a corresponding continual secretion of bile (33). Consequently, we speculate that compared with those of individuals from the captive population, the digesta of free-ranging gazelles would be characterized by higher levels of bile. Thus, the fecal concentration of hormone metabolites excreted from organisms with bile is also dependent on the speed of intestinal passage (15). In contrast, the unlimited intake of water in the captive population would facilitate a more rapid intestinal transit and evacuation together with the substances contained in the bile and thus could be a reason for the lower concentrations of fecal hormone metabolite in the captive population. Furthermore, in addition to the differences in water availability, there were notable differences between the two populations with respect to dietary intake. The diet of the captive population is consistently supplemented with hay and barley, which could influence intestinal passage (34), the free-ranging animals had no access to barley or hay. A further difference between the two populations is that within their natural habitats, free-ranging gazelles have a larger area in which to roam, potentially leading to higher levels of physical activity, which could further influence their hormone levels (35).

In contrast to testosterone, the levels of the metabolites of which showed clear differences between the two study populations, differences between the populations with respect to the fecal concentrations of progesterone metabolites were somewhat less distinct. In females, the levels of progesterone are determined to a greater extent by reproductive status than are those of testosterone. Accordingly, high levels of progesterone metabolites are to be expected during the luteal phase of the estrous cycle and pregnancy, with correspondingly lower levels during the follicular phase and anestrous phases (21, 36, 37). However, given that not all animals within a population have the same reproductive status, there would remain a high variability among individuals with respect to the synthesis of progesterone. Consequently, given these seasonal fluctuations and individual differences, the influence of any microenvironmental factors may have been obscured, with significant differences between populations only detected in July for females and in September for males (Figure 4). In this regard, it should be emphasized that, similar to the concentrations of testosterone metabolite, the aforementioned significantly higher progesterone metabolite levels were also observed in the free-ranging population.

A further finding in this study was a positive correlation between the levels of progesterone and testosterone metabolites (Figures 5, 6). During the steroidogenesis of sex hormones in steroidogenic tissues, the 21 C atoms containing progesterone is derived from the 27 C atoms containing cholesterol via the main steroid precursor, pregnenolone, facilitated by side chain cleavage catalyzed by CYP450 enzymes. Furthermore, progesterone is an intermediate in a biochemical pathway that leads (via androstenedione) to the synthesis of testosterone, which can be further converted to 17β-estradiol (12, 38). Previous studies have revealed a similar positive correlation between testosterone and progesterone levels (27, 28), indicating that the synthesis and regulation of these hormones are interlinked. The positive association between these two hormones may reflect a coordinated mechanism of hormonal regulation that supports reproductive and endocrine balance. Moreover, this correlation has been observed in different species, highlighting its potential importance in the general dynamics of reproductive physiology (39–41). Our findings in the present study are thus consistent with those previously reported, particularly in males, in which the seasonal patterns of progesterone and testosterone metabolites tend to be very similar.

Finally, our findings revealed notable seasonal variations in the levels of fecal testosterone and progesterone metabolites, which is consistent with previously reported observations of seasonal variability in the concentrations of sex hormone (42–46). However, given that this phenomenon was considered ancillary to our primary objectives, we did not undertake an in-depth examination of the causal factors in the present study. However, the raw data of fecal testosterone and progesterone metabolite concentrations are available in Supplementary Table B and can be used by readers for further statistical approaches.

A key limitation of this study is the validation of the method of fecal progesterone and testosterone metabolites detection. Since these ELISA kits were used for the first time for the detection of progesterone and testosterone metabolites in fecal samples in mountain gazelle, detailed validation is recommended (47). In this study, we conducted partial analytical validation and biological evaluation for progesterone and testosterone metabolites detection. However, we did not perform physiological validation of the detection methods, as it requires LH challenge test. For ethical and conservational reasons, the use of drugs cannot be performed on mountain gazelles as members of endangered species without clinical indication. Therefore, detailed analytical biological and physiological validations would increase the reliability of the measurements of fecal progesterone and testosterone metabolite concentrations. In addition, while the sample size for the captive population was generally sufficient across the seasons, it was relatively limited in December due to time constraints and limited accessibility to area. This may have reduced the statistical power for that time point, and we advise caution when interpreting results for this specific period.

Collectively, our findings in this study have provided evidence to indicate that the levels of testosterone metabolites in feces derived from a captive population of mountain gazelles are lower than those in the feces of free-ranging individuals. Contrastingly, we detected no clear distinction between the two populations with respect to the concentrations of progesterone metabolites. These findings are important not only for the mountain gazelle as an endangered species but also raise the question of the mechanisms underlying the dynamics of steroid hormone excretion in other ruminant species. Although we acknowledge that the explanations presented herein are not exhaustive, they do highlight potential areas for further research. In addition, our findings provide important insights into the reproductive endocrinology of mountain gazelles, which will contribute to enhancing species conservation efforts for this species and further physioecological research.

## Data Availability

The original contributions presented in the study are included in the article/Supplementary material, further inquiries can be directed to the corresponding author.

## Footnotes

^1^ https://www.r-project.org/
