# Supplementary material for: Captivity-associated variations in fecal testosterone and progesterone metabolite concentrations in mountain gazelle (Gazella gazella)
Source: Front Vet Sci. 2025 Sep 4;12:1621008. doi: 10.3389/fvets.2025.1621008 (PMC12443539; doi:10.3389/fvets.2025.1621008)
Supplement: Supplementary file 1 [file Table_1.DOCX]

**Table:** Estimated coefficients and 95% confidence intervals from the generalized linear model for fecal progesterone concentrations in *Gazella gazella,* presented on the log scale. The reference group is Female, April, Captive.

| **Predictor** | **Estimate** | **Lower 95% CI** | **Upper 95% CI** |
| --- | --- | --- | --- |
| (Intercept) | 7.418 | 6.626 | 8.493 |
| Season [July] | -1.992 | -3.202 | -0.937 |
| Season [September] | -0.490 | -1.691 | 0.547 |
| Season [December] | 0.294 | -1.371 | 2.557 |
| Captivity [FR] | 0.150 | -1.094 | 1.280 |
| Sex [M] | 0.140 | -1.061 | 1.177 |
| Sex [M] : Season [July] | 0.313 | -1.065 | 1.794 |
| Sex [M] : Season [September] | -1.690 | -3.117 | -0.134 |
| Sex [M] : Season [December] | -1.850 | -4.290 | 0.143 |
| Sex [M] : Captivity [FR] | -0.260 | -1.674 | 1.229 |
| Season [July] : Captivity [FR] | 1.077 | -0.291 | 2.509 |
| Season [September] : Captivity [FR] | -0.798 | -2.154 | 0.626 |
| Season [December] : Captivity [FR] | -0.136 | -2.539 | 1.754 |
| Sex [M] : Season [July] : Captivity [FR] | -0.351 | -2.187 | 1.435 |
| Sex [M] : Season [September] : Captivity [FR] | 2.165 | 0.281 | 3.978 |
| Sex [M] : Season [December] : Captivity [FR] | 1.139 | -1.222 | 3.849 |

Estimates and 95% confidence intervals are presented on the log scale. The reference group is Female, April, Captive.

CI – 95% confidence interval

FR – Free-ranging

M – Male

**Table:** Estimated coefficients and 95% confidence intervals from the generalized linear model for fecal testosterone concentrations in *Gazella gazella,* presented on the log scale. The reference group is Female, April, Captive.

| **Predictor** | **Estimate** | **Lower 95% CI** | **Upper 95% CI** |
| --- | --- | --- | --- |
| (Intercept) | 5.468 | 5.132 | 5.847 |
| Season [July] | -0.476 | -0.920 | -0.055 |
| Season [September] | -0.083 | -0.522 | 0.333 |
| Season [December] | -0.491 | -1.169 | 0.272 |
| Captivity [FR] | 0.842 | 0.398 | 1.263 |
| Sex [M] | 0.159 | -0.280 | 0.575 |
| Season [July] : Captivity [FR] | -0.346 | -0.862 | 0.182 |
| Season [September] : Captivity [FR] | -0.862 | -1.374 | -0.337 |
| Season [December] : Captivity [FR] | -0.151 | -0.975 | 0.599 |
| Season [July] : Sex [M] | -0.066 | -0.611 | 0.493 |
| Season [September] : Sex [M] | -0.391 | -0.957 | 0.195 |
| Season [December] : Sex [M] | -0.232 | -1.088 | 0.563 |
| Captivity [FR] : Sex [M] | -0.637 | -1.174 | -0.086 |
| Season [July] : Captivity [FR] : Sex [M] | 0.659 | -0.036 | 1.344 |
| Season [September] : Captivity [FR] : Sex [M] | 1.135 | 0.424 | 1.833 |
| Season [December] : Captivity [FR] : Sex [M] | 0.660 | -0.268 | 1.634 |

Estimates and 95% confidence intervals are presented on the log scale. The reference group is Female, April, Captive.

CI – 95% confidence interval

FR – Free-ranging

M – Male
